# The Role of Socioeconomic Background in Career Choice and Perceptions of the Graduate Nursing Residency Program Among Nursing Students in Hungary

**DOI:** 10.3390/nursrep16080270

**Published:** 2026-08-03

**Authors:** Edit Nyergesné Závodi, Viktor Simon, Krisztina Antónia Bornemissza, Borbála Mikos, György János Velkey, Gusztáv Róbert Stubnya, Helga Judit Feith

**Affiliations:** 1 Health Sciences Division, Doctoral College, Semmelweis University, 1088 Budapest, Hungary; nyergesne.edit@phd.semmelweis.hu (E.N.Z.); simon.viktor@bethesda.hu (V.S.); 2 Bethesda Children’s Hospital, 1146 Budapest, Hungary; mikos.borbala@bethesda.hu (B.M.); foigazgato@bethesda.hu (G.J.V.); 3 Faculty of Economics, Health Sciences and Social Studies, Károli Gáspár University of the Reformed Church in Hungary, 1131 Budapest, Hungary; stubnya.gusztav.robert@kre.hu; 4 Faculty of Humanities and Social Sciences, Péter Pázmány Catholic University, 1111 Budapest, Hungary; bornemissza.krisztina.job@gmail.com; 5 Department of Social Sciences, Faculty of Health Sciences, Semmelweis University, 1088 Budapest, Hungary

**Keywords:** Bachelor of Science in Nursing students, career choice, social background, socialization, student background types, graduate nursing residency program, professional integration

## Abstract

**Introduction:** The current shortage in the global nursing workforce underscores the importance of understanding factors associated with career choice and professional retention. This study aimed to identify background profiles among Hungarian Bachelor of Science in Nursing students and to examine their associations with career-choice patterns and perceptions of the Graduate Nursing Residency Program (GNRP). **Methods:** A cross-sectional online survey was conducted between 1 September 2025 and 1 March 2026 at seven Hungarian higher education institutions. Of 979 questionnaires screened, eight were excluded because of incomplete cluster-analysis data, resulting in an analytical sample of 971 students. Student background profiles were explored using TwoStep cluster analysis. The four-cluster solution was retained on the basis of model-fit change, cluster-size balance, parsimony, and substantive interpretability; the average silhouette value was 0.20, indicating weak separation. **Results:** Four exploratory profiles were identified: students from families with a university degree (17.3%), students with a rural healthcare orientation (37.3%), urban middle-class students (24.2%), and students with an urban mobility background (21.2%). The timing of career choice differed across profiles (*p* < 0.001; Cramer’s V = 0.179). Awareness of the GNRP was limited, with 69.7% reporting no prior knowledge. Perceived contribution to professional development differed statistically across profiles (*p* = 0.033; partial η^2^ = 0.009), but the effect was small. **Conclusions:** The findings describe exploratory background profiles and modest associations rather than stable student categories or causal effects. Limited awareness and generally favorable perceptions of the GNRP suggest potential relevance for future communication and evaluation, but the present study does not establish program effectiveness.

## 1. Introduction

Healthcare systems face significant human resource challenges. One of the most serious of these is the shortage of nursing staff [1]. Educating a new generation of nurses, supporting their retention in the profession, and facilitating the professional integration of young professionals have therefore become tasks of paramount importance [2]. According to the international literature, retaining the nursing workforce and ensuring the successful professional integration of new graduates are among the most significant human resource challenges in healthcare today [2,3]. Among newly graduated nurses, turnover rate exceeds 33% two years after graduation, which leads to staff shortages in care teams and has a negative impact on the quality of care [4,5].

International literature increasingly emphasizes that the transition from nursing education to clinical practice is a critical period for newly graduated nurses, during which professional identity, perceived competence, and organizational support strongly influence early career development and retention. Previous studies have shown that career choice in nursing is shaped by both altruistic and pragmatic motivations, including willingness to help others, job security, family and social influences, professional development opportunities, and perceptions of the social status of the profession [6]. In this context, structured transition and residency programs have gained international attention as potential strategies to support professional integration and reduce early career uncertainty [7].

According to the international literature, choosing a career in nursing is a complex process in which altruistic motivations, job security, career opportunities, and characteristics of one’s social and family background all play a role [8,9]. According to recent research, supportive attitudes continue to play a decisive role in career choices, and, in addition, job security, opportunities for professional development, and social recognition also emerge as significant motivating factors [10,11,12]. Numerous studies have shown that parents’ educational attainment, the family’s socioeconomic status, the type of community in which these families live, and family members’ educational background can have a significant impact on the choice of higher education studies and the development of professional identity [13,14]. Social background and the socialization environment can play a significant role in shaping educational and professional trajectories. Family resources, the local community, school experiences, and past socialization patterns can equally influence individuals’ professional aspirations, career choices and future career plans [15,16]. The choice of a career in healthcare should therefore not be viewed solely as the result of individual preferences, but also as a process that is shaped by one’s social environment and the experiences gained throughout one’s life [17].

The theoretical framework of the present study is based on three complementary perspectives. First, social determinants of educational and career trajectories suggest that family background, parental education, place of residence, and previous educational pathways may influence access to higher education and professional aspirations. Second, career choice theory highlights the interaction between individual motivations, perceived opportunities, and social influences in shaping career decisions. Third, professional identity frameworks emphasize that the development of a nursing identity begins before entry into clinical practice [18,19,20] and continues during education and the transition to the workplace [18]. On this basis, the present study examines whether distinct social and educational background profiles can be identified among Bachelor of Science in Nursing students and whether these profiles are associated with career choice patterns and perceptions of the Graduate Nursing Residency Program (GNRP).

However, students enrolled in nursing programs cannot be regarded as a homogeneous group. Differences in family background, community environments, prior educational experiences, and socialization patterns can lead to the formation of distinct student groups [14]. These differences may be reflected not only in the timing and motivations behind career choices but also in attitudes towards professional development, career building, and programs designed to support those starting their careers [8,9,21]. In recent years, the role of graduate nurses has gained greater recognition worldwide. As healthcare becomes more complex, interprofessional collaboration strengthens, and the demand for higher levels of clinical competence increases, there is a growing emphasis on supporting the professional development of graduate nurses [22]. The development and strengthening of professional identity is particularly important during the transition from higher education to the workforce, as it can significantly influence career retention and professional commitment [14,21].

The transition from nursing student to clinical practice is a complex, multifaceted process in which new nurses often face significant challenges adapting to their new role [4]. A sense of low competence, emotional strain, and a need for support often lead to “transition shock,” which can have long-term effects on the future careers of new nurses. This results in reduced job satisfaction, low retention rates, and higher rates of neglecting nursing duties [4]. To address these challenges, many countries have introduced residency or transitional professional programs designed to ease the transition from higher education to the workforce and to reduce the risk of dropping out [23,24]. The nursing residency program is a structured transitional program designed to develop the competencies of newly graduated nurses and to facilitate their transition into professional practice through mentoring, support, and hands-on experience [4,25]. Residency programs typically last one or two years, although there are also shorter and longer options [5]. The programs generally include several key components: (a) a preceptor-guided orientation period, during which new nurses are introduced to the patient care unit under the guidance of an experienced nurse; (b) a formal mentoring system that provides both professional and emotional support; (c) structured competency development to acquire basic and specialty-specific competencies; (d) regular reflective seminars where new graduates can share their experiences; and (e) a clinical rotation system across various patient care units [5,26].

According to research on residency programs, such initiatives can significantly contribute to the development of professional competencies, increased self-confidence, stronger organizational integration, and longer-term retention in the profession [23,24,27,28]. Edwards et al. and Rush et al. reported that structured transition and residency programs may contribute to improved competence, self-confidence, job satisfaction, organizational integration, and retention among newly graduated nurses [4,5]. Participation in one-year residency programs may reduce turnover and increase retention, with some programs reporting first-year retention rates as high as 87% [26,29]. Professional support for entry-level nurses is particularly important given that, among recent graduates, the intention to leave the profession is a significant problem even at the international level [3,28].

Although previous international studies have examined nursing career choice, professional identity formation, and the transition from education to practice, less is known about how students from different social and educational backgrounds perceive structured graduate nursing residency programs before entering the workforce. This gap is particularly relevant in Central and Eastern European healthcare systems, where nursing workforce shortages and retention challenges are substantial, but residency-type support programs are still emerging. Addressing this gap may help educators, healthcare institutions, and policymakers design more targeted communication and support strategies for nursing students and newly graduated nurses. The aim of this study was to explore what student groups can be distinguished based on the social and socialization characteristics of Bachelor of Science in Nursing students in Hungary. The study also examined how these groups differ in their nursing career choice patterns, perceptions of occupational prestige, awareness of the GNRP, and evaluations of the program’s potential contribution to professional development and labor market integration.

## 2. Materials and Methods

### 2.1. Study Design

The study was conducted as a quantitative, cross-sectional study using a self-administered online questionnaire. The aim of the data collection was to identify the social, educational, and socialization backgrounds of Bachelor of Science in Nursing students, to characterize career-choice patterns associated with these backgrounds, and to examine how these backgrounds relate to awareness of and perceptions regarding the Graduate Nursing Residency Program (GNRP).

### 2.2. Participants and Setting

The target population consisted of students enrolled in Bachelor of Science in Nursing programs at the seven participating Hungarian higher education institutions. Students were eligible if they were enrolled in one of these programs during the data-collection period. According to verified institutional enrolment data, the eligible population comprised 2261 students. A total of 979 submitted questionnaires were screened, corresponding to 43.3% of the eligible population. Eight respondents were excluded because they had incomplete data on variables required for the cluster analysis, resulting in a final analytical sample of 971 participants, representing 42.9% of the eligible population. No statistical imputation was performed. Complete data on all cluster variables were required for cluster allocation, whereas subsequent analyses used available valid responses on an analysis-specific basis. Although the analytical sample covered a substantial proportion of the eligible population and included students from all seven institutions, participation was voluntary and uneven across institutions. Therefore, the sample should be regarded as a large multicentre convenience sample rather than as a statistically representative sample of all Hungarian Bachelor of Science in Nursing students.

### 2.3. Recruitment and Data Collection

Data collection was conducted online between 1 September 2025 and 1 March 2026. Students were approached through institutional communication channels made available by the participating universities. The invitation described the purpose of the study, voluntary participation, anonymity, and access to the questionnaire. Although individual receipt or opening of the invitation was not tracked, 979 questionnaires were submitted, corresponding to an overall participation proportion of 43.3% of the eligible population. Participation varied considerably across institutions, with institutional sample sizes ranging from 17 to 295 respondents. This uneven participation, together with voluntary participation and the high proportions of part-time students and students already employed in nursing, limits conclusions regarding national representativeness. Individual questionnaire completion times were not available for analysis; based on the length and structure of the instrument, the expected completion time was approximately 15–20 min.

### 2.4. Questionnaire Development and Measures

The questionnaire comprised 39 numbered questions, several of which contained multi-item rating matrices. It was organized into thematic sections covering sociodemographic and educational background, career-choice motivations, parental support, perceived occupational prestige, awareness of the GNRP, and perceptions of the program’s structural and professional elements. Items used categorical response options, multiple-choice formats, and four- or five-point rating scales. Examples include questions on the timing of career choice, the influence of job security, and the perceived contribution of the GNRP to professional development. The instrument was reviewed for content relevance by experts in nursing education, health sciences, and social science methodology. The GNRP attitude scale demonstrated high internal consistency (Cronbach’s α = 0.872). The questionnaire items used in the present analyses, including their full wording and response options, are provided in Supplementary File S1.

The GNRP refers to the structured transition program examined in this study. Occupational prestige refers to students’ perceptions of the social standing of the nursing profession, whereas perceptions of the GNRP refer to students’ evaluation of the residency program and its potential role in professional development.

### 2.5. Statistical Analysis

Data analysis was performed using IBM Statistical Package for the Social Sciences (SPSS) Statistics 25. Descriptive statistics included frequency distributions, percentages, means, and standard deviations.

Before analysis, data were screened for invalid values, inconsistent response patterns, and missing information. Of 979 questionnaires screened, eight were excluded because at least one variable required for cluster allocation was missing; the cluster analysis therefore used *n* = 971 complete cases. No missing values were imputed. For all other descriptive and inferential analyses, available-case analysis was used, and percentages were calculated from valid responses for the relevant item. Accordingly, valid sample sizes varied slightly: the cluster analysis and core sociodemographic descriptions used *n* = 971; the categorical career-choice analyses reported in Table 1 used *n* = 957–971 depending on the item; analyses of GNRP awareness and general perceptions used *n* = 971; and the MANOVA and related structural-element analyses used *n* = 937 complete cases. The valid sample size is reported in the relevant table note or results paragraph. Selected categorical variables were recoded or collapsed into analytically meaningful categories to avoid sparse cells and support interpretable cluster formation. The coding procedures are presented in Table 2.

TwoStep cluster analysis was selected because the aim was to explore naturally occurring student background profiles based on the joint distribution of categorical and ordinal social, educational, and socialization variables. Candidate solutions were examined using the log-likelihood distance measure. Cluster selection was not based on a single statistic; four complementary criteria were considered: (1) change in Schwarz’s Bayesian Information Criterion (BIC) and the ratio of distance measures, (2) avoidance of very small clusters, (3) balance of cluster sizes, and (4) parsimony and substantive interpretability of the resulting profiles. Although the automatic BIC procedure suggested six clusters, the fixed four-cluster solution was retained because it preserved a substantial BIC reduction of 484.656, had a BIC-change ratio of 0.567 and a distance-measure ratio of 1.182, produced four non-trivial clusters ranging from 168 to 362 students (17.3–37.3%; largest-to-smallest ratio = 2.15), and yielded coherent profiles defined by distinguishable combinations of parental education, residence, schooling, and healthcare orientation. The additional complexity of the six-cluster solution was not considered justified by improved substantive interpretability. The average silhouette measure for the four-cluster model was 0.20, indicating weak cohesion and separation. The retained clusters were therefore treated as exploratory descriptive profiles, not as a definitive or externally validated classification.

After identifying student background profiles, associations between cluster membership and categorical career-choice variables were examined using Pearson’s chi-square tests. Cramer’s V was calculated to estimate the strength of associations. All significant and non-significant chi-square findings are reported with the chi-square statistic, degrees of freedom, exact *p*-value, and Cramer’s V effect size.

Before ANOVA and MANOVA, the distributions of the dependent variables and the relevant assumptions were examined. Normality was assessed using distributional indicators and graphical inspection, homogeneity of variances was evaluated using Levene’s test, and equality of covariance matrices was assessed using Box’s M test. Given the large sample size and the relative robustness of ANOVA and MANOVA to moderate deviations from normality, parametric analyses were considered appropriate for the rating-scale outcomes. Levene’s tests indicated unequal variances for selected outcomes, which were therefore interpreted cautiously. Box’s M was significant (Box’s M = 130.908; F = 2.052; *p* < 0.001), indicating violation of covariance-matrix homogeneity; consequently, Pillai’s Trace was used to interpret the multivariate effect.

MANOVA was selected because the examined GNRP components represented several conceptually related dependent variables. The multivariate approach allowed the combined pattern of differences across student background profiles to be evaluated in a single model and reduced the risk of inflated Type I error associated with conducting multiple separate ANOVAs. Pillai’s Trace was used for interpreting multivariate effects because it is considered robust in larger samples.

In the analyses, the level of statistical significance was set at *p* < 0.05. When interpreting the results, attention was paid to effect sizes, particularly Cramer’s V and partial eta-squared measures.

### 2.6. Ethical Considerations

The research was approved by the Scientific and Research Ethics Committee of the Health Sciences Council (case identification number: BM/23058-3/2024). Participation in the study was voluntary and anonymous. Prior to completing the questionnaire, participants received information about the purpose of the research, the methods of data handling, and the conditions of participation. Informed consent was obtained from all participants before questionnaire completion. The research was conducted in accordance with the ethical principles of the Declaration of Helsinki.

## 3. Results

### 3.1. Characteristics of the Study Sample

The final analysis sample consisted of 971 nursing students enrolled in BSc programs. Of the respondents, 85.68% were female, and 14.32% were male. The largest age group consisted of students aged 18–24 (36.35%), while the 25–34 (22.76%) and 45–54 (22.04%) age groups were also represented in significant proportions.

Of the 971 participants, 73.43% were enrolled in part-time programs and 68.80% were already working in nursing. Institutional participation was uneven: Semmelweis University contributed 286 respondents, Károli Gáspár University of the Reformed Church in Hungary 295, the University of Pécs 31, the University of Miskolc 104, Széchenyi István University 147, the University of Szeged 17, and the University of Debrecen 91. The final analytical sample represented 42.9% of the verified eligible population of 2261 students. The majority of participants lived in urban areas, and secondary or vocational education was the most common parental educational background. These distributions characterize the participating sample. However, because participation was voluntary and institutional contributions were uneven, they should not be interpreted as demonstrating national representativeness.

Table 3 summarizes the key sociodemographic and educational characteristics of the analytical sample. Detailed item wording is provided in the Supplementary Material.

### 3.2. Profile of Student Background Types

Table 4 presents the percentage distribution of selected social, educational, and socialization characteristics within each student background profile. The table should be interpreted as a profile description: higher percentages indicate characteristics that are more typical of a given cluster, whereas lower percentages indicate less typical features. The four profiles therefore represent different combinations of family educational background, place of residence, educational pathway, and previous socialization experiences.

The first cluster (*n* = 168; 17.30%) can be identified primarily as a group of students from families with a university degree. The defining feature of this profile is the high level of parental educational attainment, as 76.19% of fathers and 83.93% of mothers had a university degree. This profile therefore represents students with higher family cultural resources. Other residential and educational characteristics should be interpreted as complementary profile features rather than as the basis of the cluster label.

The second cluster (*n* = 362; 37.28%) can be interpreted as a group with a rural healthcare orientation. The profile is characterized by lower parental educational attainment and a stronger connection to vocational or healthcare-related educational pathways. The label was retained because the cluster combines social-background indicators with educational characteristics that suggest earlier orientation toward healthcare-related training.

The third cluster (*n* = 235; 24.20%) can be described as an urban middle-class student profile. This group is characterized mainly by parental educational backgrounds concentrated at vocational-secondary or secondary levels and by a mixed but comparatively urban educational pathway. The cluster label therefore refers to the combination of secondary-level family educational resources and urban educational or residential characteristics rather than to a single background variable.

The fourth cluster (*n* = 206; 21.22%) can be identified as a group with an urban mobility background. In this manuscript, the term “urban mobility background” refers to students with predominantly urban residence and schooling trajectories whose parents generally have vocational or secondary education rather than university degrees. The label is intended to reflect an urban educational pathway combined with potential intergenerational educational mobility, rather than geographic mobility alone. This cluster is also characterized by a near-total absence of religious education, as only 1.94% of its members reported participation in religious education during high school.

### 3.3. Career Choice Patterns and Occupational Prestige by Student Background Type

To provide a more in-depth characterization of student background types, cross-tabulation analyses were used to examine differences among clusters in the timing of choosing the nursing profession, motivations for career choice, and perceptions of the nursing profession’s social prestige.

The stage of life at which students chose the nursing profession showed a significant difference across student background types (χ^2^ (6) = 62.523; *p* < 0.001; Cramer’s V = 0.179). The proportion of students who had already oriented towards the nursing profession during elementary school or earlier was the highest among students with healthcare orientation living in rural areas (44.20%), while the majority of students from families with a college education background (61.90%) decided to pursue nursing after high school graduation.

The perception of job security showed a significant correlation with cluster membership (χ^2^ (6) = 23.422; *p* = 0.001; Cramer’s V = 0.110). Job security was the most prevalent among students with healthcare orientation living in rural areas (60.83%).

Social prestige, as a factor of career choice, also showed a significant difference between the clusters (χ^2^ (6) = 24.090; *p* = 0.001; Cramer’s V = 0.112). 24.02% of students with healthcare orientation living in rural areas identified this as an influencing factor.

The role of earning potential also showed a significant association with cluster membership (χ^2^ (6) = 22.277; *p* = 0.001; Cramer’s V = 0.108). This motivation was the most prevalent among students with healthcare orientation living in rural areas (20.95%).

Perceptions of the current social prestige of the graduate nursing profession also differed significantly across student background types (χ^2^ (9) = 28.111; *p* = 0.001; Cramer’s V = 0.098). More positive perceptions of prestige were more common among students with healthcare orientation living in rural areas and those with an urban mobility background.

For the non-significant associations, complete statistical reporting is provided in Table 1. The clusters did not differ substantially in terms of the influence of parents and acquaintances, the presence of healthcare workers among family or friends, career opportunities, the role of academic grades, the motivation to help people, parental support, or perceptions of future prestige; however, these null findings should be interpreted together with the reported χ^2^ statistics, degrees of freedom, exact *p*-values, and Cramer’s V effect sizes.

Overall, the statistically significant associations were modest in magnitude (Cramer’s V = 0.098–0.179). They should therefore be interpreted as exploratory differences within the responding sample rather than as evidence that social or educational background strongly determines career choice or occupational perceptions.

### 3.4. Awareness and Perception of the GNRP

There was no significant association between student background profile and prior awareness of the GNRP (χ^2^(3) = 1.394; *p* = 0.707; Cramer’s V = 0.038). The proportion of students who had previously heard of the program was similar across all clusters (28–33%).

Among the 971 valid respondents, 69.72% reported that they had no prior information about the GNRP.

The high proportion reporting no prior information indicates limited awareness of the GNRP within the responding sample. However, the cross-sectional survey did not assess students’ opportunities to receive information or the effectiveness of institutional communication; the finding should therefore not be interpreted as direct evidence of inadequate dissemination. All analyses in Section 3.4 used *n* = 971 valid responses.

Perceptions of the program’s role in supporting professional development differed significantly across clusters (F(3, 967) = 2.933; *p* = 0.033; partial η^2^ = 0.009). Students from families with a university-degree background rated the program’s professional usefulness more positively than students from urban middle-class and urban mobility backgrounds.

A significant difference was also observed in the assessment of the program’s prestige-enhancing effect (F(3, 967) = 2.841; *p* = 0.037; partial η^2^ = 0.009), although post hoc comparisons did not clearly identify distinct cluster pairs.

No significant differences were found between background profiles in the assessment of the program’s innovativeness (F(3, 967) = 0.454; *p* = 0.715; partial η^2^ = 0.001), organizational acceptance (F(3, 967) = 0.510; *p* = 0.675; partial η^2^ = 0.002), or professional support (F(3, 967) = 0.556; *p* = 0.644; partial η^2^ = 0.002).

### 3.5. Student Background Types and Perceptions of the Structural Elements of the GNRP

The complete-case MANOVA included 937 participants; 34 of the 971 students were excluded from this analysis because at least one of the structural-element ratings was missing. The multivariate association between cluster membership and the combined evaluation of program characteristics was statistically significant (Pillai’s Trace = 0.044; F(18, 2790) = 2.311; *p* = 0.001; partial η^2^ = 0.015).

Despite statistical significance, the multivariate effect size was small (partial η^2^ = 0.015). This result indicates limited practical differences among the exploratory profiles and should not be interpreted as evidence that background profile has a strong influence on program evaluations.

In the *n* = 937 complete-case sample, the only statistically significant univariate difference concerned the rotation system (F(3, 933) = 5.242; *p* = 0.001; partial η^2^ = 0.017). Students from families with a university-degree background reported stronger agreement that the three-month rotation could help resident nurses become familiar with hospital patient-care units. The effect was small, and the finding should be interpreted as an exploratory difference in perception rather than evidence of differential program benefit.

No significant differences were observed among the student background groups in their assessment of the program’s other features, including professional mentoring, spiritual mentoring, theoretical continuing education, the two-year training period, and broader integration into the hospital’s organizational culture and professional environment. Complete univariate results for the non-significant outcomes are reported as follows: professional mentoring (F(3, 933) = 2.447; *p* = 0.063; partial η^2^ = 0.008), spiritual mentoring (F(3, 933) = 1.508; *p* = 0.211; partial η^2^ = 0.005), theoretical continuing education (F(3, 933) = 0.350; *p* = 0.789; partial η^2^ = 0.001), the two-year training period (F(3, 933) = 0.273; *p* = 0.845; partial η^2^ = 0.001), and broader integration into the hospital’s organizational culture and professional environment (F(3, 933) = 0.388; *p* = 0.761; partial η^2^ = 0.001).

## 4. Discussion

The aim of this study was to explore what student groups can be distinguished based on the social and socialization characteristics of Bachelor of Science in Nursing students in Hungary. The study also examined how these groups differ in their patterns of nursing career choice, perceptions of occupational prestige, awareness of the GNRP, and evaluations of the program’s potential contribution to professional development and labor market integration. The results indicate that the student population examined cannot be considered homogeneous but consists of distinct subgroups with different social and educational backgrounds.

The findings suggest that social background does not simply describe student characteristics, but may also shape the timing and perceived meaning of nursing career choice. However, the generally small effect sizes indicate that these differences should not be interpreted deterministically. Rather, social and educational background should be understood as a set of contextual factors that interact with individual motivation, educational experience, and professional expectations. This interpretation is consistent with international research emphasizing that nursing career choice and professional identity formation are dynamic processes shaped by both personal and sociocultural influences.

Although several differences between student background profiles reached statistical significance, the effect sizes were generally small. These findings therefore indicate modest associations rather than strong or deterministic effects. Social and educational background may contribute to differences in career-choice trajectories and perceptions of the GNRP, but the practical importance of these associations should be interpreted cautiously and in combination with other individual, educational, and organizational factors.

The cluster analysis identified four distinct student background types, among which the group of students with healthcare orientation living in rural areas is particularly noteworthy. This cluster not only had the largest number of participants but also exhibited a distinctive pattern across several indicators of career choice. Members of this group were more likely to have decided on a nursing career at a young age, and they more frequently cited job security, social recognition, and earning potential as motivations for their career choice. Our findings are consistent with those of Eley and colleagues, who found that entering the nursing profession is motivated by both altruistic and pragmatic factors [9]. Lommi and colleagues, who studied Generation Z students, also pointed out that, in addition to willingness to help others, job security and opportunities for career advancement play an increasingly important role in career choices [10]. Lin and colleagues’ longitudinal qualitative study provided further evidence that the formation of professional identity is a dynamic process influenced by both internal motivations and external sociocultural factors [30].

In addition, our findings also revealed that social background is linked not only to the timing of career choice but also to the social perception of the profession. Students with a healthcare orientation living in rural areas and those with an urban mobility background rated the current prestige of the graduate nursing profession more positively than members of other clusters did [31]. This finding is consistent with the research of Johnson et al. and Lommi et al., which suggests that professional identity and perceptions of the profession’s social standing are closely linked [10,13]. A more positive perception of the profession’s social value can contribute to greater professional commitment and retention, which is particularly important in the context of the current global nursing shortage [30].

Prior awareness of the GNRP was similarly limited across the four exploratory profiles, and nearly 70% of respondents reported no previous knowledge of the program. This finding describes awareness in the participating sample but does not establish why awareness was low or whether existing communication was ineffective. The result nevertheless identifies a question for future research on how and when nursing students encounter information about structured transition programs.

Respondents generally evaluated the described GNRP positively, and most ratings did not differ meaningfully among profiles. These ratings reflect students’ perceptions of a program description at one point in time; they do not demonstrate that participation in the GNRP improves competence, job satisfaction, integration, or retention. The consistency with international studies of implemented transition programs provides contextual support for further evaluation, but it should not be interpreted as evidence of effectiveness in the present sample.

The clearest profile difference concerned the rotation system, which students from university-educated families rated somewhat more favorably; however, the effect size was small (partial η^2^ = 0.017). Explanations relating this difference to stronger emphasis on professional development remain speculative because such mechanisms were not directly tested. The finding should therefore be considered hypothesis-generating and examined in future qualitative or longitudinal research.

### 4.1. Practical Implications

The findings may help educators and program designers consider the social and educational diversity of nursing students when planning communication about residency or transition support. Because the profiles were exploratory and effect sizes were small, the results do not justify rigid segmentation of students or profile-specific interventions without further validation. They instead suggest that clear, accessible information about mentoring, rotation, professional development, and institutional integration may be relevant across student groups.

Future implementation studies could examine whether providing information about the GNRP during nursing education, clinical-practice orientation, career guidance, or recruitment changes awareness, perceived relevance, or subsequent participation. The present cross-sectional data support testing such communication strategies but cannot determine that they will increase uptake or improve retention.

### 4.2. Limitations

Several limitations should be acknowledged. First, the cross-sectional design supports description of associations only and does not permit causal inference or assessment of changes over time. Second, all measures were self-reported and may be affected by recall error, social-desirability bias, and differences in interpretation of questionnaire items. Third, recruitment was conducted through voluntary, anonymous institutional communication channels. Although verified institutional enrolment data indicated an eligible population of 2261 students, individual receipt or opening of the invitation was not tracked. Therefore, the 979 submitted questionnaires, corresponding to 43.3% of the eligible population, represent an overall participation proportion rather than a conventional individually tracked response rate. Participation was uneven across institutions, and the sample contained high proportions of part-time students and students already employed in nursing. Consequently, national representativeness cannot be assumed. Voluntary participation, self-selection, non-response bias, and differences in participation across institutions may have influenced the composition of the sample and the observed findings. Fourth, the cluster analysis was data-driven and exploratory. Although the automatic procedure suggested six clusters, a four-cluster solution was retained using statistical, size-balance, parsimony, and interpretability criteria. The silhouette value of 0.20 indicated weak separation, cluster labels involved substantive interpretation, and the solution was not tested in an independent or split sample; therefore, the profiles should not be treated as stable or validated categories. Fifth, no imputation was performed and available-case analysis resulted in slightly different sample sizes across analyses. If missingness was systematic, this approach may have introduced additional bias. Finally, the study assessed awareness and perceptions of the GNRP rather than actual participation or outcomes, and it did not examine the reasons for limited awareness. Accordingly, the findings do not demonstrate program effectiveness, effects on retention, or the effectiveness of any proposed dissemination strategy.

## 5. Conclusions

The exploratory analysis identified four background profiles within this multicenter sample of Hungarian Bachelor of Science in Nursing students. Because cluster separation was weak and the sample’s representativeness could not be established, these profiles should be understood as descriptive patterns in the present data rather than as fixed or nationally generalizable student categories.

Social and educational background showed small associations with selected career-choice patterns and perceptions. These observations may help generate hypotheses about the contexts in which nursing career decisions develop, but they do not show that background characteristics cause the observed differences.

Awareness of the GNRP was limited across the exploratory profiles, while evaluations of the described program were generally favorable. These findings provide a rationale for further testing of communication and transition-support strategies during nursing education, but they do not establish that targeted dissemination will increase participation or that the GNRP will improve workforce retention.

Future research should replicate and validate the cluster solution in independent samples, compare the sample with verified national enrolment data, and use longitudinal designs to follow students into early-career practice. Qualitative studies could also examine reasons for limited GNRP awareness and how students understand individual program components.

The study provides exploratory multicenter evidence on the diversity of nursing students’ backgrounds, career-choice patterns, and perceptions of a graduate nursing residency program in Hungary. Its main contribution is to identify questions and tentative patterns for subsequent confirmatory and longitudinal investigation.

## Figures and Tables

**Table 1:** Career choice patterns and occupational prestige in the total sample.

Variable	Option	Sample (N)	Ratio (%)	Association by Student Background Type
**Timing of career choice** (valid *n* = 971)	Elementary school age or younger	325	33.47	**χ^2^(6) = 62.523; *p* < 0.001;** **Cramer’s V = 0.179;** **significant**
	High school age	236	24.30	
	After graduating from high school	410	42.22	
**Career choice motivation: parents/acquaintances** (valid *n* = 957)	Did not influence at all	500	52.25	**χ^2^(6) = 8.285; *p* = 0.218;** **Cramer’s V = 0.066;** **non-significant**
	Influenced to a lesser extent	238	24.87	
	Influenced	219	22.88	
**Career choice motivation: healthcare workers in family/friends** (valid *n* = 961)	Did not influence at all	567	59.00	**χ^2^(6) = 7.240; *p* = 0.299;** **Cramer’s V = 0.061;** **non-significant**
	Influenced to a lesser extent	180	18.73	
	Influenced	214	22.27	
**Career choice motivation: career opportunities** (valid *n* = 961)	Did not influence at all	377	39.23	**χ^2^(6) = 9.337; *p* = 0.155;** **Cramer’s V = 0.070;** **non-significant**
	Influenced to a lesser extent	308	32.05	
	Influenced	276	28.72	
**Career choice motivation: academic grades** (valid *n* = 957)	Did not influence at all	660	68.97	**χ^2^(6) = 6.276; *p* = 0.393;** **Cramer’s V = 0.057;** **non-significant**
	Had little influence	159	16.61	
	Had influence	138	14.42	
**Career choice motivation: helping people** (valid *n* = 962)	Had no influence at all	47	4.89	**χ^2^(6) = 9.807; *p* = 0.133;** **Cramer’s V = 0.071;** **non-significant**
	Had little influence	135	14.03	
	Had influence	780	81.08	
**Career choice motivation: job security** (valid *n* = 967)	Had no influence at all	162	16.75	**χ^2^(6) = 23.422; *p* = 0.001;** **Cramer’s V = 0.110;** **significant**
	Had little influence	271	28.02	
	Had influence	534	55.22	
**Career choice motivation: social recognition** (valid *n* = 960)	Had no influence at all	440	45.83	**χ^2^(6) = 24.090; *p* = 0.001;** **Cramer’s V = 0.112;** **significant**
	Had little influence	340	35.42	
	Had influence	180	18.75	
**Career choice motivation: earning potential** (valid *n* = 959)	Had no influence at all	427	44.53	**χ^2^(6) = 22.277; *p* = 0.001;** **Cramer’s V = 0.108;** **significant**
	Had little influence	375	39.10	
	Had influence	157	16.37	
**Parental support** (valid *n* = 971)	Opposed the election	136	14.01	**χ^2^(3) = 6.528; *p* = 0.089;** **Cramer’s V = 0.082;** **non-significant**
	Supported the election	835	85.99	
**Perceived occupational prestige** (valid *n* = 971)	Very low	148	15.24	**χ^2^(9) = 28.111; *p* = 0.001;** **Cramer’s V = 0.098;** **significant**
	Somewhat lower	503	51.80	
	Somewhat higher	309	31.82	
	Very high	11	1.13	
**Expected future prestige** (valid *n* = 971)	No	209	21.52	**χ^2^(6) = 10.999; *p* = 0.088;** **Cramer’s V = 0.075;** **non-significant**
	Yes	387	39.86	
	Don’t know/No answer	375	38.62	

Note. Concise variable labels are used to improve readability; full survey item wording is provided in Supplementary File S1. Percentages are based on valid responses, and no missing values were imputed. Statistical test results, exact *p*-values, and Cramer’s V are reported for both significant and non-significant associations.

**Table 2:** Variables included in the TwoStep cluster analysis and their analytical coding.

Variable	Original Response Categories	Analytical Coding/Categorisation	Notes
Father’s education	Fewer than 8 grades of primary education; 8 grades of primary education; vocational training school; secondary school; college; university	Collapsed into: primary or less; vocational school; vocational secondary school; university degree	Used as a social-background indicator in the TwoStep cluster analysis.
Mother’s education	Fewer than 8 grades of primary education; 8 grades of primary education; vocational training school; secondary school; college; university	Collapsed into: primary or less; vocational school; vocational secondary school; university degree	Used as a social-background indicator in the TwoStep cluster analysis.
Current place of residence	Budapest; county seat; city; town; village	Recoded into: capital city; county seat; town/city; village	Used to characterize current settlement background.
Location of elementary school	Budapest; county seat; town; village/town	Recoded into: capital city; county seat; town; village	Used to represent earlier educational and settlement background.
Type of secondary school	General high school; healthcare-focused high school; healthcare technical school; healthcare vocational high school; non-healthcare vocational/specialized/technical school; other	Collapsed into broader secondary-school pathway categories relevant to nursing career orientation	Used to capture previous educational pathway.
Religious education during high school	No; yes, for less than a semester; yes, for more than a semester	Collapsed into: no participation; participation	Used as a socialization-related indicator.

**Table 3:** Sociodemographic and educational characteristics of the study sample.

		Sample (N)	Ratio (%)
**Gender**	Men	139	14.32
	Women	832	85.68
**Age**	Aged 18–24	353	36.35
	Aged 25–34	221	22.76
	Aged 35–44	165	16.99
	Aged 45–54	214	22.04
	55 and older	18	1.85
**Marital status**	Single and not in a relationship	316	32.54
	Single and in a domestic partnership	225	23.17
	Married, living with a spouse	311	32.03
	Divorced/separated and living alone	48	4.94
	Divorced/separated, but in a domestic partnership	39	4.02
	Other (e.g., in a relationship, widowed, engaged, etc.)	32	3.30
**Institution of study**	Semmelweis University	286	29.45
	Károli Gáspár University of the Reformed Church in Hungary	295	30.38
	University of Pécs	31	3.19
	University of Miskolc	104	10.71
	Széchenyi University	147	15.14
	University of Szeged	17	1.75
	University of Debrecen	91	9.37
**Year of study**	First	339	34.91
	Second	251	25.85
	Third	310	31.93
	Fourth	71	7.31
**Program type**	Full–time	258	26.57
	Part–time	713	73.43
**Current nursing employment**	No	303	31.20
	Yes	668	68.80
**Current residence**	Budapest	280	28.84
	County seat	134	13.80
	City	330	33.99
	Town	219	22.55
	Village	8	0.82
**Father’s education**	Fewer than 8 grades of primary education	6	0.62
	8 grades of primary education	81	8.34
	Vocational Training School	450	46.34
	Secondary school	243	25.03
	College	88	9.06
	University	103	10.61
**Mother’s education**	Fewer than 8 grades of primary education	6	0.62
	8 grades of primary education	109	11.23
	Vocational Training School	286	29.45
	Secondary school	356	36.66
	College	105	10.81
	University	109	11.23
**Location of elementary school**	Budapest	182	18.74
	County seat	126	12.98
	Town	390	40.16
	Village/Town	273	28.12
**Type of secondary school**	High school	455	46.86
	Healthcare-focused high school	104	10.71
	Healthcare technical school	61	6.28
	Healthcare vocational high school	191	19.67
	Non-healthcare-specialized vocational high school, specialized high school, technical school	157	16.17
	Other	3	0.31
**Religious education during** **high school**	No	651	67.04
	Yes, for less than a semester	27	2.78
	Yes, for more than a semester	293	30.18

**Table 4:** Student background profiles identified by TwoStep cluster analysis: percentage distribution of selected characteristics within each cluster. The automatic TwoStep procedure suggested a six-cluster solution. The four-cluster solution was retained after considering model-fit change together with cluster-size adequacy, balance, parsimony, and substantive interpretability. For the four-cluster solution, BIC was 10,996.241, the BIC reduction was 484.656, the ratio of BIC changes was 0.567, and the ratio of distance measures was 1.182. Cluster sizes ranged from 168 students (17.3%) to 362 students (37.3%), with a largest-to-smallest ratio of 2.15, and each profile could be interpreted using a coherent combination of family educational background, residence, schooling, and healthcare orientation.

Variable	Category	1-Students from Families with a University Degree (*n* = 168; 17.3%)	2-Students with a Rural Healthcare Orientation (*n* = 362; 37.3%)	3-Urban Middle-Class Students (*n* = 235; 24.2%)	4-Students with an Urban Mobility Background (*n* = 206; 21.2%)
Father’s education	Primary or less	0	17.96	5.11	4.85
	Vocational school	6.55	66.57	42.13	48.06
	Vocational secondary school	17.26	14.36	41.28	31.55
	University degree	76.19	1.1	11.49	15.53
Mother’s education	Primary or less	0.6	20.44	8.51	9.71
	Vocational school	1.79	44.75	23.4	32.04
	Vocational secondary school	13.69	29.83	60.43	40.29
	University degree	83.93	4.97	7.66	17.96
Place of residence	**Capital city**	43.45	14.64	51.91	15.53
	County seat	14.29	9.94	30.21	1.46
	**Town/city**	16.67	30.39	11.06	80.58
	**Village**	25.6	45.03	6.81	2.43
**Location of elementary school**	**Capital city**	57.14	4.97	82.55	0
	Town	26.19	34.53	8.94	97.09
	**Village**	16.67	60.5	8.51	2.91
**Type of secondary school**	**Grammar school**	88.69	35.91	36.17	44.17
	**Healthcare vocational secondary school**	7.14	48.34	42.98	33.01
	**Non-healthcare vocational/technical school**	4.17	15.75	20.85	22.82
**Religious education during high school**	**No participation**	44.05	48.9	84.26	98.06
	**Participation**	55.95	51.1	15.74	1.94

Note. Values represent percentages within each cluster and should be interpreted as profile characteristics rather than as mean scores or participant counts. The cluster headings include both the sample size and percentage of the total sample. Higher percentages indicate characteristics that are more typical of a given cluster, whereas lower percentages indicate less typical features. The color scale indicates the relative proportion of each category within the given cluster: green indicates a relatively high proportion, amber indicates a moderate proportion, and red indicates a relatively low proportion.

## Data Availability

The datasets generated and analyzed during the current study are available from the corresponding author on reasonable request. The data are not publicly available due to ethical and privacy considerations.

## Supplementary Materials

The following supporting information can be downloaded at: https://www.mdpi.com/article/10.3390/nursrep16080270/s1. Supplementary File S1 contains an English translation of all questionnaire items and response options used in the analyses reported in this manuscript.

## Public Involvement Statement

No public involvement in any aspect of this research.

## Guidelines and Standards Statement

This manuscript was drafted in accordance with the Strengthening the Reporting of Observational Studies in Epidemiology (STROBE) Statement for cross-sectional observational research [32].
