# Supplementary material for: The Role of Socioeconomic Background in Career Choice and Perceptions of the Graduate Nursing Residency Program Among Nursing Students in Hungary
Source: Nurs Rep. 2026 Aug 3;16(8):270. doi: 10.3390/nursrep16080270 (PMC13516105; doi:10.3390/nursrep16080270)
Supplement: Supplementary file 1 [file nursrep-16-00270-s001.zip › nursrep-4406545-supplementary.pdf]

Supplementary file

Questionnaire Items Included in the Analyses

The Role of Socioeconomic Background in Career Choice and Perceptions of the Graduate Nursing Residency Program Among Nursing Students in Hungary

Edit Nyergesné Závodi; Viktor Simon; Krisztina Antónia Bornemissza; Borbála Mikos; György János Velkey; Gusztáv Róbert Stubnya; Helga Judit Feith

Scope of this supplementary file. The original survey contained 39 numbered questions, several with multi-item rating matrices. This document reproduces the items and response options included in the descriptive and inferential analyses reported in the manuscript. Original questionnaire numbering is retained; items not analyzed in the present study are not reproduced.

S1. Sociodemographic and Educational Background Items

1. At which institution are you pursuing your nursing studies?
Response options: Semmelweis University; Károli Gáspár University of the Reformed Church in Hungary; University of Pécs; University of Miskolc; Széchenyi István University; University of Szeged; University of Debrecen.

2. What year of the nursing program are you currently in?
Response options: First year; Second year; Third year; Fourth year.

3. Which type of study program are you completing?
Response options: Full-time; Part-time.

4. Are you currently working as a nurse?
Response options: Yes; No.

5. What is your age?
Response options: Open numerical response in completed years.

6. What is your gender?
Response options: Man; Woman.

7. Where do you currently live? (Excluding the location of your studies.)
Response options: Budapest; County seat; City; Town; Village.

8. What is your father's highest level of education?
Response options: Fewer than 8 grades of primary education; 8 grades of primary education; Vocational training school; Secondary school; College; University.

9. What is your mother's highest level of education?
Response options: Fewer than 8 grades of primary education; 8 grades of primary education; Vocational training school; Secondary school; College; University.

10. Where did you complete your elementary school education?
Response options: Budapest; County seat; Town/City; Village.

12. At what type of school did you earn your high school diploma?
Response options: General high school; Healthcare-focused high school; Healthcare technical school; Healthcare vocational high school; Non-healthcare vocational/specialized/technical school; Other.

13. Did you receive religious education during your high school studies?
Response options: No; Yes, for less than one semester; Yes, for more than one semester.

14. What is your marital status?
Response options: Single and not living with a partner; Single and living with a partner; Married and living with a spouse; Divorced/separated and living alone; Divorced/separated but living with a partner; Other.

S2. Career Choice and Occupational Prestige Items

15. At what stage of your life did you decide to become a nurse?
Response options: Elementary school age or younger; High school age; After graduating from high school.

16. How did the following factors influence your choice of career?
Response scale: 1 = Had no influence at all; 2 = Had little influence; 3 = Had influence.

Item	Statement
1	Influence of parents and acquaintances.
2	There are healthcare workers in my family and circle of friends.
3	Career opportunities in the field.
4	I was accepted into the nursing program based on my academic grades/admission score.
5	I enjoy helping people and working with them.
6	Job security.
7	Social recognition.
8	Earning potential.

17. What was your parents' opinion of your career choice?
Response options: They opposed my choice; They supported my choice.

18. In your opinion, what is the social prestige of the nursing profession?
Response options: Very low; Somewhat low; Somewhat high; Very high.

19. Do you think the prestige of the nursing profession will increase in the future?
Response options: Yes; No; I do not know.

S3. Graduate Nursing Residency Program Items

Program description presented to respondents. Before answering the following items, participants received a description of the two-year Graduate Nursing Residency Program implemented at Bethesda Children's Hospital. The description explained that newly graduated nurses rotate through the hospital's patient-care units according to a predefined schedule; participate in theoretical training covering quality improvement, patient safety, patient documentation, communication, and institution-specific topics; and receive regular professional and spiritual mentoring to support their integration, clinical development, and reflection on workplace experiences.

28. Were you aware of the nurse residency program before completing this questionnaire?
Response options: Yes; No.

29. What do you think of the program described above in terms of professional development for nurses?

Response options: Does not seem useful at all; Does not seem very useful; Seems useful; Seems very useful.

31. In your opinion, how would the nationwide implementation of this program affect the status and prestige of the nursing profession?

Response options: It would worsen; It would not change; It would improve.

32. Please rate each feature of the program individually.

Response scale: 1 = Do not think it is good at all; 2 = Do not really think it is good; 3 = Tend to think it is good; 4 = Think it is very good.

Item	Statement
1	Through the three-month rotation system, resident nurses become familiar with all of the hospital's patient-care units.
2	The residency program lasts two years.
3	Professional mentoring for resident nurses.
4	Spiritual mentoring for resident nurses.
5	Theoretical continuing education during the program.
6	Over the course of two years, resident nurses become familiar with a broad range of hospital staff.

35. Please indicate the extent to which you agree with each statement about the program.

Response scale: 1 = Strongly disagree; 2 = Somewhat disagree; 3 = Agree; 4 = Strongly agree.

Item	Statement
1	I consider this program to be an innovative initiative that offers a new way to address the critical shortage of nurses.
2	The resident nurse program increases the chances of retaining nurses.
3	Nurses working in patient-care units are willing to welcome resident nurses and assist with their training and integration.
4	Nurses working in patient-care units supervise and train resident nurses.

Note. This supplementary file provides an English translation of the original Hungarian questionnaire items used in the analyses. The original question ***numbering, response structure, and meaning of the response options have been retained. Terminology has been harmonized with that used in the revised manuscript.***
